# Severe Postpartum Hemorrhage Complicated with Liver Infarction Resulting in Hepatic Failure Necessitating Liver Transplantation

**DOI:** 10.1155/2018/2794374

**Published:** 2018-06-11

**Authors:** I-Ting Peng, Ming-Ting Chung, Ching-Chung Lin

**Affiliations:** ^1^Department of Obstetrics and Gynecology, Chi Mei Medical Center, Taiwan; ^2^Chia Nan University of Pharmacy & Science, Taiwan

## Abstract

Postpartum hemorrhage remains a major threat to maternal health. Intervention after critical blood loss or development of disseminated intravascular coagulation may lead to disastrous organ failure and poor outcomes. A 30-year-old woman was transferred to our emergency department due to massive postpartum hemorrhage. Shock and disseminated intravascular coagulation ensued, and the patient's condition quickly deteriorated. We performed an emergency hysterectomy, but blood loss had been massive. Moreover, there was another episode of internal bleeding that led to further blood loss. Ischemic injury to the liver was tremendous, with resulting progressive jaundice and hepatic encephalopathy. The patient required liver transplantation. Imaging studies and operative findings showed a large area of hepatic infarction. Unfortunately, the patient died of intractable sepsis shortly after liver transplantation. Disseminated intravascular coagulation and resultant hepatic infarction combined with ischemic hepatitis were the direct cause of death in our case.

## 1. Introduction

Hemorrhage is the leading cause of maternal mortality in developing and developed countries [1]. Although several risk factors are associated with postpartum hemorrhage (PPH), it is still impossible to predict it. As a result, early recognition of PPH and prompt action to curtail it are crucial to a good outcome. If we do not stop the bleeding at an early stage, severe consequences including disseminated intravascular coagulation (DIC) and organ failure can develop. We present a case of severe PPH complicated by DIC and hepatic infarction resulting in acute hepatic failure. The patient did not survive even after liver transplantation and died on the 36th day after the primary PPH episode.

## 2. Case Presentation

**Figure 1:**
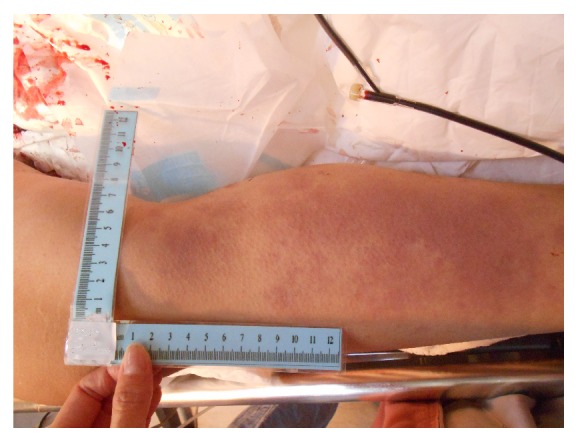
Ecchymosis on calves developed as one of the signs of DIC after uncontrollable PPH.

A 30-year-old woman was transferred to our emergency department five hours after delivering her baby at a clinic. She was a primipara at 41 weeks of gestation. She delivered a baby with vertex presentation vaginally, without dystocia. Massive vaginal bleeding started 2 hours after delivery. After excluding birth canal laceration and retaining placental tissue, the obstetrician began IV fluid and uterotonic treatment, but the bleeding continued. She was then transferred to our hospital due to PPH. However, when she arrived, she had severe tachycardia (heart rate, 160 bpm) and hypotension (BP, 44/34 mmHg). Her consciousness was clear, but she was agitated. We immediately began transfusion of packed red blood cells (6 units), fresh frozen plasma (4 units), apheresis platelets (2 units), and whole blood (2 units) as we simultaneously examined the patient. Signs of DIC developed with continuous blood loss (Figure 1), and her consciousness deteriorated within 30 minutes after arriving at the emergency department.

Uterine atony and an ischemic uterus were found during emergency laparotomy. A subtotal hysterectomy was completed. Intraoperative blood loss was 800 mL. The patient was transferred to the ICU after surgery. Her postoperative fibrinogen level was 54.6 mg/dL (normal: 200–400 mg/dL). We transfused fresh frozen plasma and cryoprecipitate to achieve a fibrinogen level greater than 100 mg/dL. However, unstable blood pressure and progressive abdominal distension were found 4 hours after the primary surgery. We rushed the patient back into surgery due to suspicion of internal bleeding. Hemoperitoneum of 2000 mL and active bleeding from ruptured pararectal vessels were identified. After the secondary surgery for ligation of the bleeding vessels, the patient had acute kidney injury with anuria, intractable hyperkalemia, and metabolic acidosis. Thus she underwent continuous venovenous hemofiltration (CVVH).

The patient's hemodynamic status and ventilation function gradually improved after hemostasis. CVVH was shifted to intermittent hemodialysis on postoperative day 10. She was extubated on the same day.

**Figure 2:**
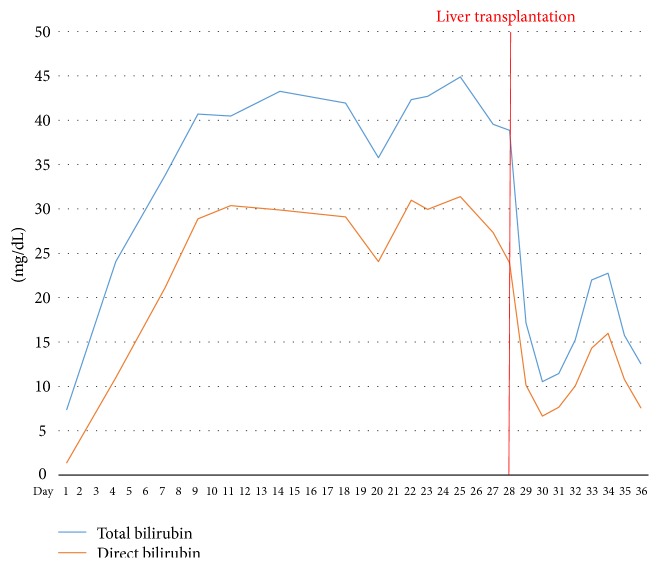
Trend of bilirubin levels.

**Figure 3:**
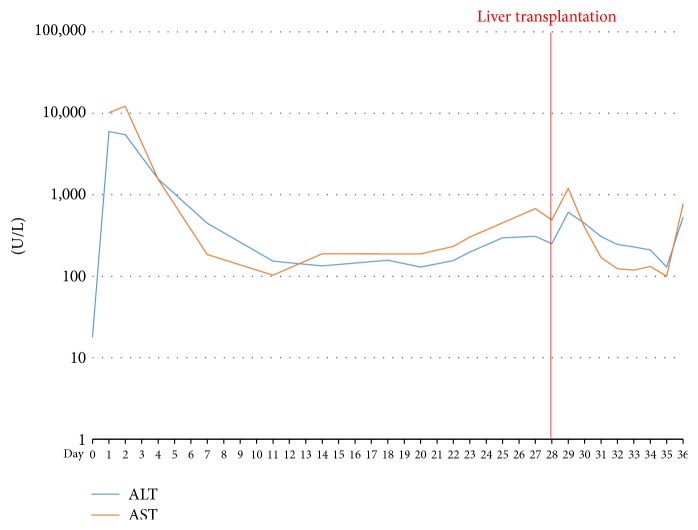
Trend of AST/ALT levels.

**Figure 4:**
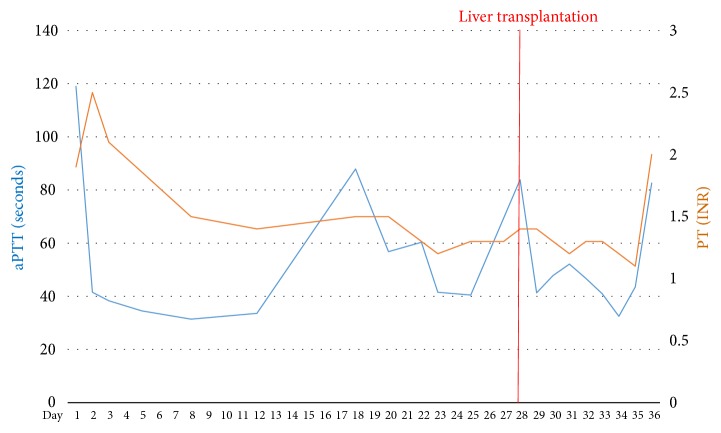
Trend of PT/aPTT.

Unfortunately, hyperbilirubinemia progressed and became the main problem (Figure 2). Liver enzyme levels peaked on postoperative day 3 and then settled to about 100–200 IU/L (Figure 3). Thrombocytopenia continued, along with prolonged prothrombin time (INR, 1.2–1.5) and activated partial thromboplastin time (1.5–2.5 times of normal control) (Figure 4). Abdominal ultrasonography revealed no biliary tract obstruction but an ill-defined hypoechoic lesion in the right lobe of the liver, about 6.5 × 6.5 cm, probably due to an inflammatory process or tumor growth. Abdominal CT was indicated to confirm the characteristic of the lesion but was postponed due to the patient's poor renal function. After consultation with a gastrointestinal expert, the lesion was thought to be a liver abscess or focal necrosis due to ischemic change. Her consciousness had been deteriorating since post-PPH day 20 in combination with intractable fever. Brain CT showed no intracranial lesion. Worsening cognition was likely due to metabolic encephalopathy of hepatic or infectious origin.

**Figure 5:**
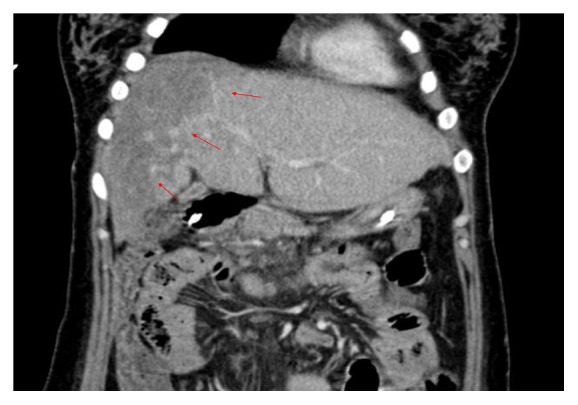
Pretransplantation CT scan showed a large area of infarction in the right lobe of the liver (arrows).

**Figure 6:**
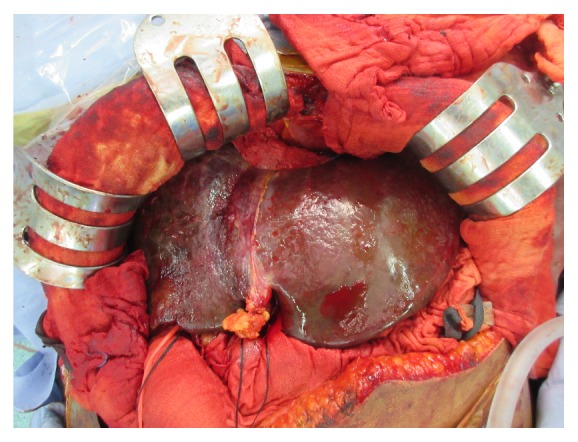
Gross appearance of the patient's liver during liver transplantation

**Figure 7:**
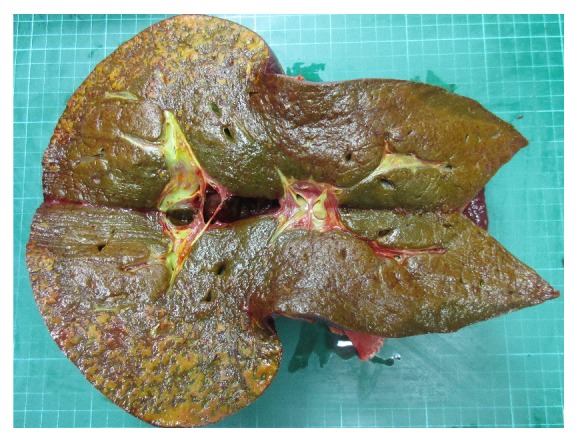
Transection of the resected native liver (1850 g).

A liver transplantation was indicated, and a pretransplantation CT scan showed poor enhancement of the right hepatic lobe on the periphery covering about 50% of the total area, which was suggestive of liver infarction (Figure 5). Living donor liver transplantation was performed 28 days after PPH. The donor was her younger brother, and the graft liver weight was 840 gm. The graft-to-recipient weight ratio was 1.24%. Intraoperatively, there were multiple micronodular lesions on the liver surface with marked swelling of the liver parenchyma and diffuse devitalized liver tissue in the right lobe of liver with necrosis and in the left lobe peripheral zone accounting for about 70%–80 % of the total liver volume (Figures 6 and 7).

The patient's bilirubin level improved on the first three days but increased again on the 4th day after transplantation. The coagulation parameters did not change significantly after liver transplantation. Serial liver ultrasonography showed acceptable vascular status of the transplanted liver, without thrombosis or biliary obstruction. Fever, tachycardia, and hypotension occurred on the 6th day after transplantation as well as vaginal and anal stool leakage. An emergency colostomy and perianal debridement were done under the suspicion of a perianal abscess and rectovaginal fistula. Unfortunately, the patient died of an intractable infection the next day.

## 3. Discussion

Maternal mortality rate (MMR) is an important index of fetomaternal health. WHO announced in 2016 that 99% of all maternal deaths occur in developing countries. MMR in developing countries in 2015 was 239 per 100 000 live births versus 12 per 100 000 live births in developed countries. The MMR in Taiwan in 2015 was 11.7 per 100 000 live births. There were 25 maternal deaths reported in 2015. This is a relatively low MMR compared to other areas worldwide. However, this number could be underestimated [2]. Up to two-thirds of all maternal deaths would be missed if we relied on the death registration only [3]. In addition, the true incidence of other obstetric complications is even harder to calculate accurately. For example, severe preeclampsia complicated with aortic dissection or peripartum cardiomyopathy might be erroneously linked to hypertensive cardiovascular disease but not gestation-related. Likewise, PPH may cause multiple organ failure, including acute renal failure, stroke, and ischemic liver failure, but the incidence of these complications is unknown.

In a previous report, a patient with severe PPH experienced an acute myocardial infarction on the 7th postpartum day [4]. She also had ischemic hepatitis, and her AST and ALT levels peaked on the 3rd day after PPH (her liver enzyme trend was similar to that of our patient). Her liver function then recovered spontaneously after correction of hypoperfusion and left no obvious sequela. In contrast, our patient experienced serious complications, including intractable hepatic failure. What is the difference between the two cases? We hypothesize that the large area of hepatic infarction might have played a pivotal role in our patient.

Ischemic hepatitis and hepatic infarction are two different mechanisms of vascular injury to liver tissue. Ischemic hepatitis involves the whole liver. It is caused by systemic hypoperfusion, or it may be caused by decreased perfusion from the hepatic artery and/or portal vein. For example, it can occur in patients who undergo liver transplantation and have hepatic artery thrombosis. It is mostly a self-limiting disease, and the only effective treatment is to remove the insult that caused the liver hypoperfusion. In contrast, hepatic infarction represents focal ischemic injury to the liver. Most of the time this infarction is caused by occlusion of an intrahepatic branch of the hepatic artery. It may occur accidentally during ligation of the hepatic artery during surgery, after hepatic artery chemoembolization, or in a hypercoagulable state, with related thrombosis formation [5]. In our patient, we presumed that thrombosis formation due to DIC was the cause of the hepatic infarction. Pathologic examination of the resected liver showed small artery thrombosis. This finding supports our hypothesis. Severe ischemic hepatitis combined with a large area of hepatic infarction explains why the hepatic function of our patient remained poor even though her hemodynamic compromise had been corrected. It was difficult to predict whether or when the liver function will spontaneously recover in a patient with acute liver failure [6]. Hepatic encephalopathy, prolonged jaundice, and concomitant renal failure suggested a poor prognosis [7]. In such circumstances, the short-term mortality rate is high, even though we intervened with liver transplantation.

The timing of effective hemostasis plays a substantial role in determining a woman's prognosis after PPH. Intervening earlier when the estimated blood loss is less seems to improve outcome [8]. The PPH episode we reported occurred in an urban clinic at a distance that takes about 30 minutes by car to the nearest tertiary center. The patient had experienced significant hypotension before arrival at our center, and DIC developed shortly afterwards. The blood loss before her transfer could have been of large amount. She underwent a hysterectomy for hemostasis, yet overwhelming DIC led to the second episode of internal bleeding and hepatic infarction.

Pregnancy is a hypercoagulable status. Obstetric complications including PPH (defined as hemorrhage above 500 mL after vaginal delivery or above 1000 mL after a cesarean section), preeclampsia, hemolysis, elevated liver function tests, and low platelets (HELLP) syndrome, amniotic fluid embolism, and acute fatty liver are all conditions linked to high risk of developing DIC. A woman at 31 weeks' gestation with HELLP syndrome complicated with large area of hepatic infarction was presented in a case report [9]. This reminds us that although DIC or hepatic infarction is rare in an uncomplicated pregnant woman, we have to monitor a woman with gestational complications closely and be well prepared to detect and cope with these serious complications.

Due to the common concept of keeping the body “complete” after a person is dead among East Asian cultures, countries in this area have the lowest deceased organ donation rate in the world. In present, living donor liver transplantation (LDLT) is far more prevalent than deceased donor liver transplantation (DDLT) in Taiwan [10]. According to the data from Taiwan Organ Registry and Sharing Center, there were 406 cases of LDLT but only 108 cases of DDLT in 2017. Furthermore, there had been 1218 candidates on the waiting list for liver donation, counted until April 2018. The decision of executing liver transplantation was quick in our case, due to her worsening hepatic encephalopathy. It was hard to get a suitable cadaver donor in time. Under these circumstances, LDLT was adopted by our transplantation team. In a meta-analysis, LDLT recipients have higher short-term complication rates including biliary complications, vascular complications, and retransplantation rate compared to DDLT recipients, with a comparable perioperative mortality rate [11]. And as the transplantation team gets more experience, the outcome will be better.

One of the concerns about LDLT in our case was the following: have we got adequate graft liver transplanted? Our donor was a healthy young man, with normal BMI. A graft-to-recipient weight ratio of 0.8-1 percent is generally adequate, which in our case was 1.24 percent. The elevation of bilirubin level on posttransplantation day 4 could not be explained with inadequate size of the graft liver but rather was a presentation of early allograft dysfunction (EAD) [12]. The underlying causes of EAD include ischemia/reperfusion injury to the graft liver and acute rejection, after excluding vascular occlusion or biliary obstruction.

In conclusion, clinicians must beware of ischemic hepatitis and hepatic infarction as possible complications in victims of severe PPH. Management of these patients needs cooperation of multiple specialties and liver transplantation is the salvage treatment.
